# Deletion of the *chloroquine resistance transporter* gene confers reduced piperaquine susceptibility to the rodent malaria parasite *Plasmodium berghei*

**DOI:** 10.1128/aac.01589-24

**Published:** 2025-02-24

**Authors:** Makoto Hirai, Meiji Arai, Soki Hayamichi, Ayako Uchida, Megumi Sudo, Rie Kubota, Naoaki Shinzawa, Toshihiro Mita

**Affiliations:** 1 Department of Tropical Medicine and Parasitology, Faculty of Medicine, Juntendo University 12847 , Bunkyo-ku Hongo, Tokyo, Japan; 2 Department of International Medical Zoology, School of Medicine, Kagawa University, Kida, Kagawa, Japan; 3 Department of Parasitology and Tropical Medicine, Graduate School of Medical and Dental Sciences, Institute of Science Tokyo, Bunkyo-ku Yushima, Tokyo, Japan; The Children's Hospital of Philadelphia, Philadelphia, Pennsylvania, USA

**Keywords:** mutator, piperaquine resistance, rodent malaria parasite

## Abstract

Malaria parasites acquire drug resistance through genetic changes, the mechanisms of which remain incompletely understood. Understanding the mechanisms of drug resistance is crucial for the development of effective treatments against malaria, and for this purpose, new genetic tools are needed. In a previous study, as a forward genetic tool, we developed the rodent malaria parasite *Plasmodium berghei* mutator (PbMut) line, which has a greatly increased rate of mutation accumulation and from which we isolated a mutant with reduced susceptibility to piperaquine (PPQ). We identified a mutation in the *chloroquine resistance transporter* (PbCRT N331I) as responsible for this phenotype. In the current study, we generated a marker-free PbMut to enable further genetic manipulation of the isolated mutants. Here, we screened again for PPQ-resistant mutants in marker-free PbMut and obtained a parasite population with reduced susceptibility to PPQ. Of five isolated clones, none had the mutation PbCRT N331I; rather, they possessed a nonsense mutation at amino acid 119 (PbCRT Y119*), which would truncate the protein before eight of its ten predicted transmembrane domains. The PbCRT orthologue in the human malaria parasite *Plasmodium falciparum*, PfCRT, is an essential membrane transporter. To address the essentiality of PbCRT, we successfully deleted the full *PbCRT* gene [*PbCRT*(−)] from wild-type parasites. *PbCRT*(−) parasites exhibited reduced susceptibility to PPQ, along with compromised fitness in mice and following transmission to mosquitoes. Taken together, our findings provide the first evidence that *P. berghei* can acquire reduced PPQ susceptibility through complete loss of PbCRT function.

## INTRODUCTION

Malaria is a significant parasitic disease that poses a substantial threat to human life. The causative agents of malaria belong to the genus *Plasmodium* and are transmitted via anopheline mosquitoes. Of the human *Plasmodium* species, *Plasmodium falciparum* is the most malignant and is annually responsible for 247 million clinical cases and 608,000 deaths, mainly among children under 5 years of age residing in sub-Saharan Africa (1). Artemisinin (ART)-based combination therapies (ACTs) are used globally as first-line treatments for malaria (2). These therapies consist of fast-acting but short-lived ART derivatives in combination with longer-lasting drugs such as piperaquine (PPQ), lumefantrine, and amodiaquine. ART-resistant *P. falciparum* is emerging, as indicated by an increasing number of cases from Southeast Asia (3–5) and more recently from Africa (6, 7). As a result, the effectiveness of treatments has become increasingly reliant on partner drugs such as PPQ. However, PPQ-resistant *P. falciparum* has been documented in Southeast Asia (8), and this observation underscores the need to better comprehend the mechanisms that underlie PPQ resistance.

PPQ is composed of two 4-aminoquinoline weak-base moieties and accumulates within the acidic digestive vacuole (DV) of the malaria parasite. The antimalarial activity of PPQ is believed to be analogous to that of chloroquine in that it binds to Fe(III)-heme, which is released during the degradation of hemoglobin (Hb) in the DV. This binding inhibits the incorporation of heme into chemically inert hemozoin (Hz) crystals, which results in the accumulation of toxic heme in the parasite (9). Several studies have demonstrated that a critical role in PPQ resistance is played by the *P. falciparum* chloroquine resistance transporter (PfCRT)—a 49-kDa protein within the 10-membrane-pass transporter superfamily that is localized on the DV membrane. Various mutant isoforms of PfCRT detected in clinical isolates have been implicated in PPQ resistance. Gene editing studies have confirmed that seven PfCRT non-synonymous mutations confer PPQ resistance to transgenic parasites (10–14). In contrast, an increase in copy number of *plasmepsin II* and *III* genes has been identified as markers of PPQ resistance in isolates from Southeast Asia (15–17) although the molecular mechanism regarding how the copy number elevation results in PPQ resistance level is unclear. In the rodent malaria parasite *Plasmodium berghei*, PPQ-resistant parasites have been isolated under PPQ selective pressure (18). Subsequent study revealed no mutations in the *P. berghei chloroquine resistance transporter* (*PbCRT*) gene, while increased transcript levels were observed for the genes encoding *V-type H+-pumping pyrophosphatase (vp2*), *Ca^2+^/H+ antiporter (vcx1*), *gamma-glutamylcysteine synthetase* (*ggcs*), and *glutathione-S-transferase* (*gst)* (19). Therefore, the association of PbCRT to PPQ resistance has not been reported.

In a previous study, we reported the generation of *P. berghei* mutator (PbMut), with a mutation rate over 36-fold higher than that of wild-type parasites, which could serve as a forward genetic tool (20–22). In a subsequent study, we applied PPQ pressure to PbMut parasites to screen for PPQ-resistant parasites and successfully isolated mutants with reduced susceptibility to PPQ. Finally, we substantiated a mutation in *PbCRT* (*PbCRT* N331I) as responsible for this phenotype (23). In the present study, we utilized the CRISPR/Cas9 system to create a PbMut without a selectable marker, thereby facilitating further genetic manipulation of the mutant. We screened again for PPQ-resistant parasites in the marker-free PbMut; as a result, we isolated mutants with reduced susceptibility to PPQ and identified a novel mutation in *PbCRT*. We further demonstrated for the first time that *PbCRT* is a non-essential gene, by generating a *PbCRT* knockout parasite [*PbCRT*(−)] line. The absence of PbCRT confers reduced PPQ susceptibility to *PbCRT*(−) parasites. This phenomenon has not been observed for PfCRT.

## RESULTS

### Isolation of the mutants with reduced PPQ susceptibility from PbMut

**Fig 1:**
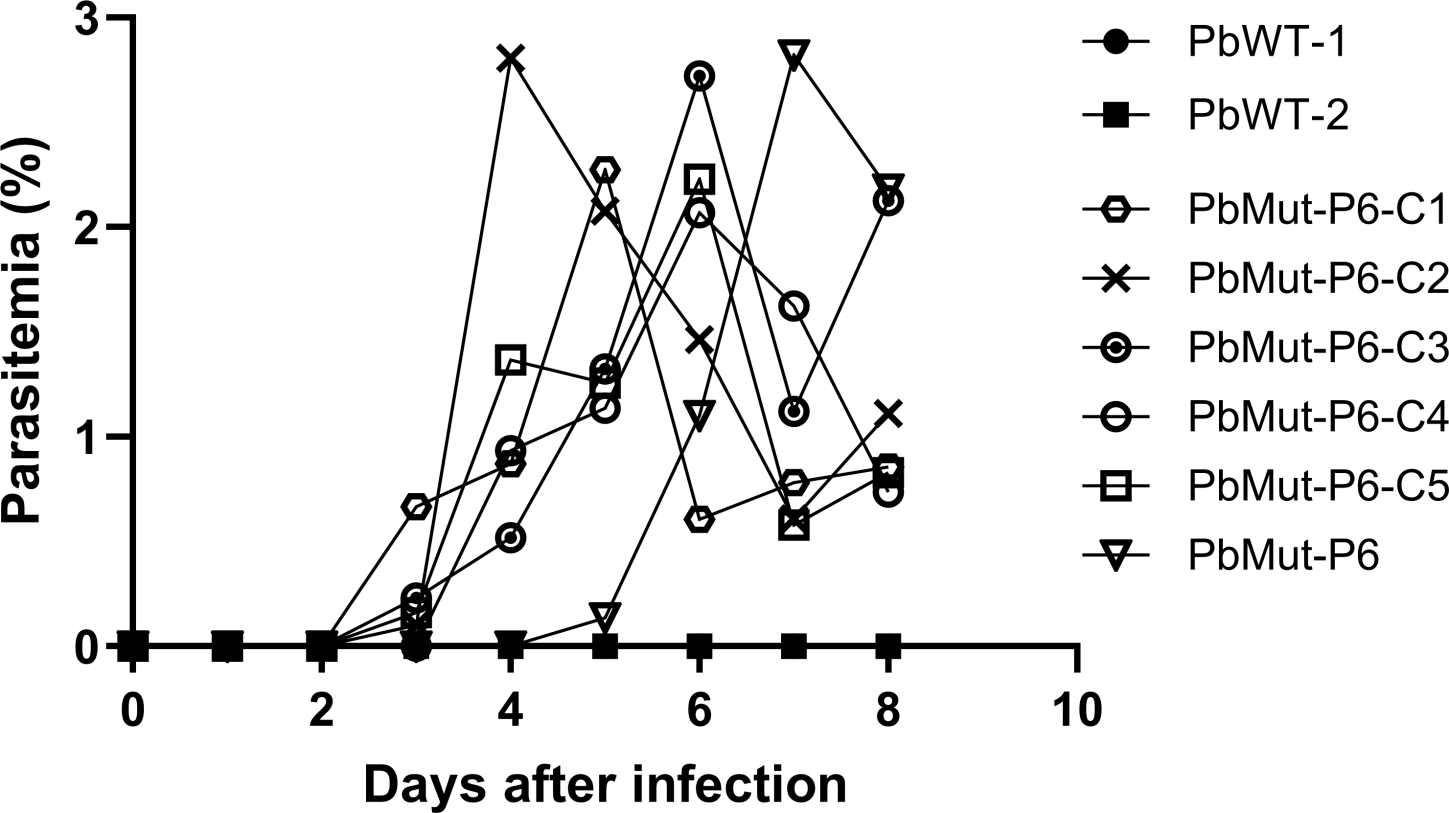
Comparison of PPQ susceptibility among PbMut-P6 clones, parental PbMut-P6, and PbWT. Mice were infected with PbMu-P6 clones (C1 to C5), parental PbMut-P6, and PbWT (clones 1 and 2) parasites and were treated with PPQ (30 mg/kg) at 4, 24, 48, and 72 h post-infection.

Mice were infected with PbMut and PbWT parasites designated as PbMut-P1 and PbWT, respectively, and were then treated with PPQ (10 mg/kg/day) for 4 days. For PbWT parasites, infected red blood cells (iRBC) were undetectable from days 7 to 19; whereas patency was detected in PbMut-P1 parasites on day 11 (Table S1). PbMut-P1 parasites (1 × 10^5^ iRBC) were transferred to a new mouse (PbMut-P2), which was treated with PPQ (20 mg/kg/day) for 6 days. Through serial passages under increasing PPQ pressure, we established lines PbMut-P1 to PbMut-P6. While the parasitemia was under the limit of detection on day 7 in PbMut-P1, parasitemia remained detectable in PbMut-P2 through PbMut-P6, even as the PPQ dose was incrementally increased from 20 mg/kg/day to 30 mg/kg/day (Table S1). Comparing the parasitemias of PbMut-P1 and PbMut-P5 on day 7, the latter showed a higher parasitemia (3.4%) than the former (0%), even though both were treated with PPQ for 4 days, with PbMut-P5 receiving 30 mg/kg/day and PbMut-P1 receiving 10 mg/kg/day (Table S1). In the last round of screening, PbMut-P6 and PbWT were treated with PPQ (30 mg/kg) at 4, 24, 48, 72, and 96 h post-infection. As a result, PbMut-P6 showed 1.3% parasitemia on day 3, while no patency was observed in PbWT until day 19 (Table S1). A comparison between PbMut-P1 and PbMut-P6 demonstrated that PbMut-P6 parasites were detectable on day 3, indicating their ability to grow under PPQ pressure. In contrast, PbMut-P1 parasites, treated with PPQ starting on day 3 when the parasitemia was 3.7%, showed parasitemia levels that fell below the limit of detection by day 7 after four consecutive days of PPQ treatment. These results suggest that mutant parasites with reduced susceptibility to PPQ emerged and were selected through six rounds of serial screening. Finally, five clones (PbMut-P6-C1 to -C5) were isolated from PbMut-P6. To evaluate the PPQ susceptibility, mice were injected with clones PbMut-P6-C1 to -C5, parental PbMut-P6, and PbWT parasites and then treated with PPQ (30 mg/kg) at 4, 24, 48, and 72 h post-infection. The parental PbMut-P6 and its clones, but not PbWT, demonstrated survival under PPQ pressure; although parasitemia levels varied among mice infected with each parasite (Fig. 1). This result indicates that each PbMut-P6 clone has acquired reduced susceptibility to PPQ.

### Single nucleotide polymorphism (SNP) analyses of the PbMut-P6 clones

To identify genetic mutations responsible for reduced susceptibility to PPQ, we performed SNP analyses on five clones, PbMut-P6-C1 to -C5, and compared them to the control clone, PbMut-P91, which was established under drug-free conditions. The number of SNPs in PbMut-P91 was much higher than that observed for PbMut-P6-C1-C5 due to the difference in passage numbers, with PbMut-P91 having a passage number of 91 and PbMut-P6-C1-C5 having a passage number of 6. As a result, a large number of SNPs conserved in PbMut-P91 were used to filter the SNPs found in the five clones, leading to the identification of 17, 17, 15, 8, and 14 new SNPs, respectively (Table S2). Among these SNPs, four non-synonymous and one nonsense mutation were conserved in all five clones (Table 1). These SNPs were in the following genes: *zinc-carboxypeptidase* (PBANKA_0209700), *sporozoite invasion-associated protein 1* (PBANKA_1006200) (24), *conserved Plasmodium protein* (PBANKA_1017800), *NAD(P) transhydrogenase* (PBANKA_1317200) (25), and *chloroquine resistance transporter* (PBANKA_1219500) (23, 26).

**TABLE 1:** Five SNPs conserved in PbMut-P6-C1 to -C5 clones with reduced PPQ susceptibility

Gene ID	Gene description	Amino acid change
PBANKA_0209700	Zinc-carboxypeptidase, putative	K1460R
PBANKA_1006200	Sporozoite invasion-associated protein 1	K896R
PBANKA_1017800	Conserved *Plasmodium* protein, unknown function	D19N
PBANKA_1219500	Chloroquine resistance transporter, putative	Tyr119*
PBANKA_1317200	NAD(P) transhydrogenase	D988N

### Generation of *PbCRT*(−) and *PfCRT* R122* parasites

**Fig 2:**
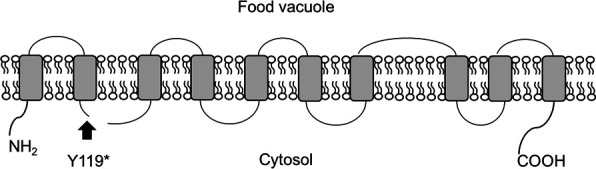
Proposed structure of PbCRT. Transmembrane domains (TM) were predicted using DeepTMHMM and are represented by gray boxes. The location of a stop-gain mutation (Y119*) is indicated in the structure by an arrow, specifically, on the cystolic side of the WT protein after the second TM domain.

Among five common SNPs detected in the PbMut-P6 clones, we focused on the SNP in *PbCRT* because it was demonstrated that the *PbCRT* mutation (PbCRT N331I) reduced susceptibility to PPQ in our previous study (23). PfCRT, the *P. falciparum* orthologue of PbCRT, has been shown to possess 10 transmembrane (TM) domains and plays a critical role in transporting molecules across the digestive vacuole (DV) (26). Analysis using DeepTMHMM predicts that PbCRT also contain 10 TM domains. The nonsense mutation at amino acid 119 is predicted to locate immediately after the second TM in PbCRT (Fig. 2). This truncation would result in the loss of roughly 70% of the protein and retention of only the first two TM domains, and it is therefore unlikely that PbCRT Y119* possesses transporter activity. However, previous studies have reported that PbCRT is vital for parasite survival because of the failure to generate a *PbCRT* gene knockout (27, 28). In this context, PbCRT Y119* may comprise a minimal essential domain that is sufficient for the transport activity required for parasite viability. Otherwise, PbCRT Y119* may be non-functional, suggesting a non-essential role of PbCRT for parasite survival.

**Fig 3:**
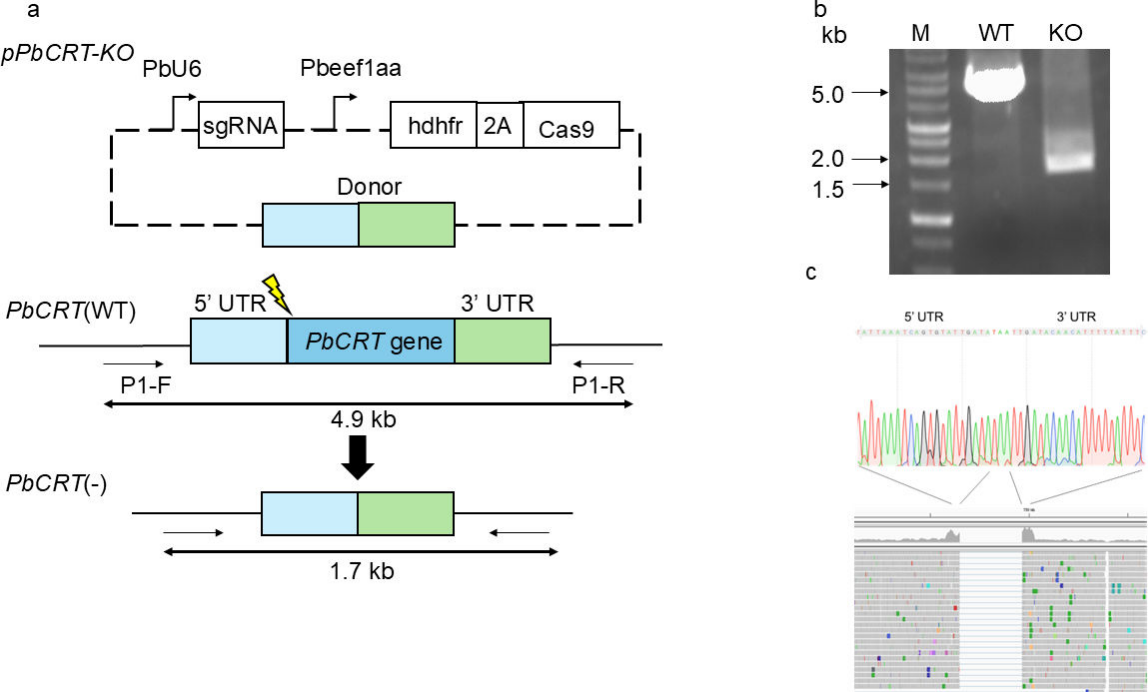
Complete deletion of the *PbCRT* gene using CRISPR/Cas9. (a) The plasmid pPbCRT-KO was used for *PbCRT* gene deletion. The sgRNA was driven by the *P. berghei* U6 promoter (PbU6). The viral “ribosome skip” 2A peptide (2A) that coordinates coexpression of the human dihydrofolate reductase (hDHFR) and Cas9 under the *elongation factor1α* promoter. The donor contains the 5′- and 3′-untranslated regions of the *PbCRT* gene. The lightning mark represents the cleavage site recognized by the sgRNA. A pair of primers, P1-F and P1-R, was used in diagnostic PCR. (b) Diagnostic PCR. Genomic DNA from wild type (WT) and *PbCRT*(−) (KO) and primers (P1-F/P1-R) were used. The expected sizes of PCR products for WT and KO were 4.9 and 1.7 kb, respectively. (c) Upper panel: Sanger sequencing data of the PCR product (1.7 kb) from *PbCRT*(−) (KO in panel b). The nucleotides indicated with light gray represent the 5′- and 3′-untranslated region of *PbCRT* gene. Lower panel: NGS analysis of a *PbCRT*(−) parasite clone. No reads are mapped to the *PbCRT* gene loci.

To determine if *PbCRT* is an essential gene, we attempted a conventional strategy to disrupt the gene with a pyrimethamine resistance marker gene; however, our repeated attempts failed (data not shown). Consequently, we employed a genome editing strategy to remove the entire *PbCRT* gene from the genome without replacing the *PbCRT* gene with a selectable marker (Fig. 3a). Diagnostic PCR using *PbCRT*(−) genomic DNA produced an amplified band of the expected size (Fig. 3b), and direct sequencing of the PCR product confirmed the presence of both the 5′ and 3′ non-coding regions but not the coding region of the *PbCRT* gene (Fig. 3c, upper panel). Whole-genome sequencing of *PbCRT*(−) was also conducted to rule out translocation of the *PbCRT* gene to other genomic regions. As a result, no reads were mapped to the *PbCRT* gene loci, demonstrating the complete deletion of the *PbCRT* gene (Fig. 3c, lower panel).

Given the successful elimination of the *PbCRT* gene, we attempted to insert a stop codon at the corresponding position in *P. falciparum* (*PfCRT* R122*) using CRISPR/Cas9-mediated genome editing (*n* = 5). After the transfection and subsequent pyrimethamine selection for 10 days, iRBC were detected on day 3 after drug removal. Genomic DNA was extracted from the parasite population on day 7, and the targeting region was amplified by PCR and the product was subjected to Sanger sequencing. As a result, however, *PfCRT* R122* was not detected in the transfected *P. falciparum* (data not shown).

### Comparison of the morphology and fitness between *PbCRT*(−) and PbWT parasites

**Fig 4:**
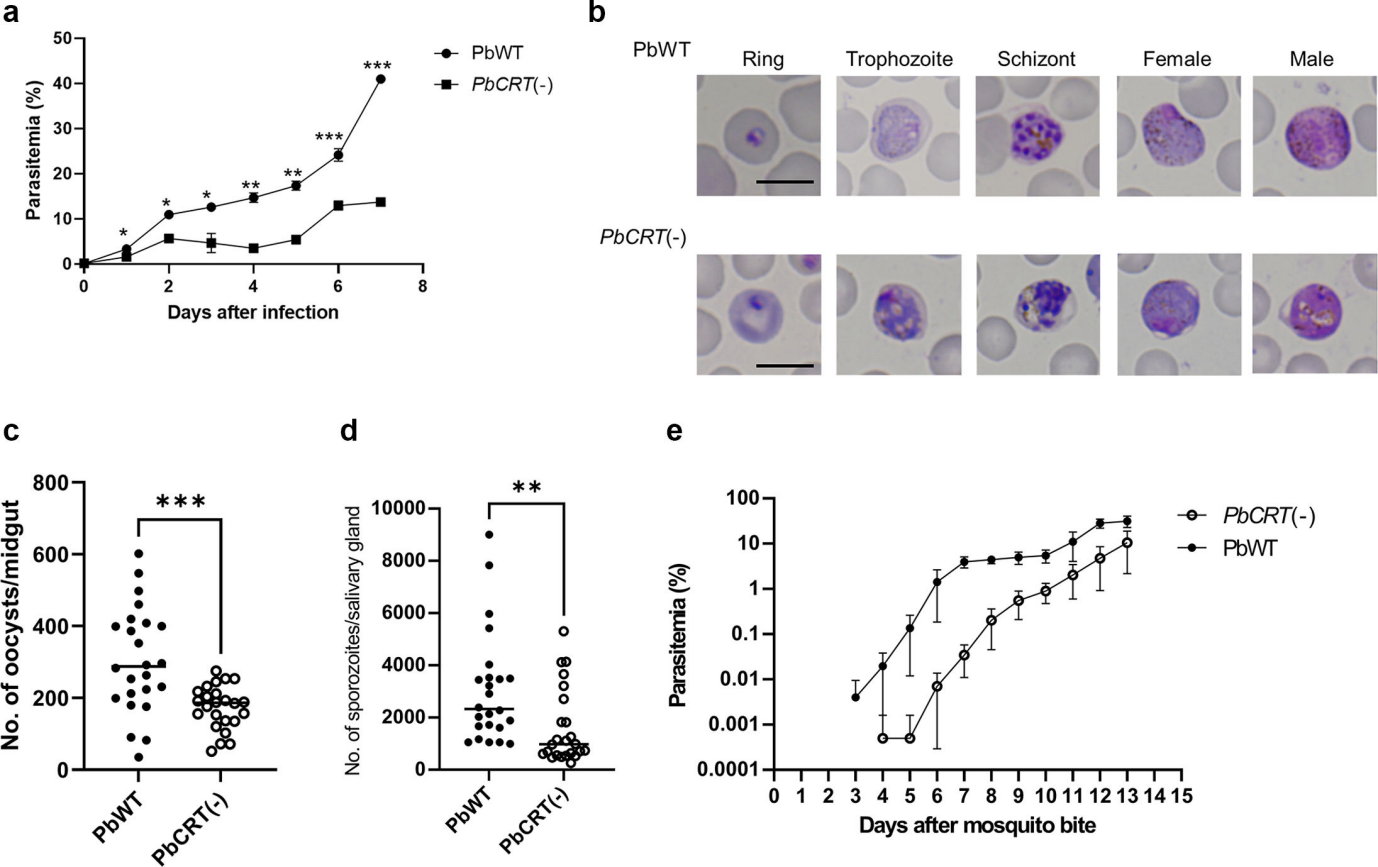
Comparison of *PbCRT*(−) parasite fitness in mouse and mosquito. (a) The parasitemias of mice infected with *PbCRT*(−) or PbWT. Erythrocytes (1 × 10^5^) infected with *PbCRT*(−) or PbWT were injected intravenously into mice, and the parasitemias were monitored until day 7 (*n* = 3, each). *P* values were generated using two-tailed Student’s *t*-test (*P < 0.05, **P < 0.01, and ***P < 0.001). (b) Comparison of cell morphology of wild-type (PbWT) and PbCRT knockout [*PbCRT*(−)] parasites. Enlarged and translucent vacuoles were observed in both asexual and sexual stages in *PbCRT*(−) parasites, with the exception of the ring stage. (c) Comparison of the numbers of oocysts on the midgut between PbWT- and *PbCRT*(−)-fed mosquitoes. The experiment was performed in triplicate. Bar represents the median oocyst number. The number of oocysts of *PbCRT*(−)-fed mosquitoes was significantly lower than that of PbWT(Mann-Whitney *U* test, *P* < 0.001). (d) Comparison of the numbers of sporozoites in the salivary gland of between PbWT- and *PbCRT*(−)-fed mosquitoes. The experiment was performed in triplicate. The bar represents the median of the sporozoite numbers in salivary glands. The number of sporozoites of *PbCRT*(−)-fed mosquitoes was significantly lower than that of PbWT (Mann-Whitney *U* test, *P* < 0.01). (e) Parasitemias of mice infected with either *PbCRT*(−) or PbWT. Mice were exposed to 25 mosquitoes carrying either *PbCRT*(−) or PbWT. *n* = 5 each.

For the assessment of fitness in mice, iRBC (1 × 10^5^) of *PbCRT*(−) and PbWT were inoculated into respective groups of three mice each, and parasitemias were monitored for 7 days. The parasitemia of *PbCRT*(−) was consistently lower than that of PbWT from days 1 to 7 (Fig. 4a). Morphological observation showed an enlarged translucent vacuole in trophozoites, schizonts, and gametocytes of *PbCRT*(−) parasites under PPQ-free conditions (Fig. 4b). Similar abnormal morphology has been seen in the PPQ-resistant PfCRT F145I parasites (14) and PfCRT knock-down parasites (29). Subsequently, the fitness of *PbCRT*(−) was assessed in mosquitoes by feeding *PbCRT*(−) and PbWT to mosquitoes. As shown in Fig. 4c, the number of *PbCRT*(−) oocysts per midgut was significantly lower than that observed for PbWT (*P* < 0.001). The number of *PbCRT*(−) sporozoites in the salivary glands was also significantly lower than that of PbWT (*P* < 0.01; Fig. 4d). To assess transmission efficiency, groups of 25 mosquitoes carrying either *PbCRT*(−) or PbWT were allowed to feed on groups of 15 mice each. The prepatent period of mice infected with *PbCRT*(−) and PbWT was 5.4 and 3.6 days, respectively, with a statistically significant difference between the two groups (*P* < 0.0001; Table 2). The subsequent parasitemia of the mice infected with *PbCRT*(−) was lower than that of PbWT (Fig. 4e). This result can be partially attributed to the lower number of *PbCRT*(−) sporozoites injected by the mosquitoes into the mice, which is due to the lower number of sporozoites present in the mosquito salivary glands compared to PbWT. Alternatively, *PbCRT*(−) may impose a fitness cost at the hepatocyte stages. Collectively, the lack of PbCRT has an impact on the fitness of both blood- and mosquito-stage parasites.

**TABLE 2:** Transmission of *PbCRT*(−) parasites to mouse^*c*^

Parasites	Infected/challenged	Pre-patency (days)^*a*^
*PbCRT*(−)	15/15	5.4^*b*^
PbWT	15/15	3.6

^*a*^ The pre-patent period represents the days until detection of the infected erythrocytes under the microscope after mosquito feeding.

^*b*^ The pre-patent period of *PbCRT*(−) is statistically different from that of PbWT (Welch's test, *P* < 0.0001).

^*c*^ The mice were exposed to 25 mosquitoes fed with *PbCRT*(−) or PbWT parasites for 6 min (1 min x 6 times).

### Assessment of PPQ susceptibility of *PbCRT*(−) parasites

**Fig 5:**
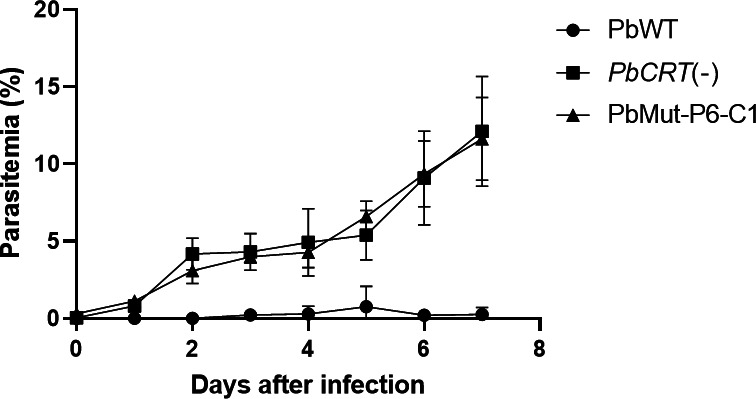
Comparison of the PPQ susceptibility among *PbCRT*(−), PbMut-P6-C1, and PbWT. Groups of three mice each were intravenously injected with iRBC (1 × 10^5^) from *PbCRT*(−), PbMut-P6-C1, and PbWT and treated with PPQ (30 mg/kg) at 4, 24, 28, and 72 h post-injection. *n* = 3 each.

The correlation of the lack of a *PbCRT* gene and PPQ susceptibility was investigated. For this, respective groups of three mice each were infected with *PbCRT*(−), PbMut-P6-C1, or PbWT and were treated with PPQ, and the resulting parasitemias were monitored. As shown in Fig. 5, both *PbCRT*(−) and PbMut-P6-C1 showed higher parasitemias than observed for PbWT. This result demonstrates that the complete deletion of *PbCRT* correlates with reduced PPQ susceptibility.

## DISCUSSION

In this study, we developed a marker-free PbMut parasite line, which we used for PPQ drug pressure to select for resistant parasites. Five parasite clones were isolated, and all possessed reduced PPQ susceptibility. Genome analysis of the clones identified five conserved SNPs, none of which were identified in our previous study (23). Possible reasons for the observation of novel SNPs might be attributed to the fact that, in this study, a marker-free PbMut that had undergone only two to three passages was used; whereas in the previous study, PbMut-P91 was used, which had undergone 91 serial passages. We reported that PbMut accumulates mutations with an increasing number of serial passages (21); therefore, the variability of the current marker-free PbMut is likely lower than that of PbMut-P91. Moreover, individual clones within PbMut-P91 were selected for their fitness over a longer passage time than marker-free PbMut, suggesting that the fitness of individual clones in marker-free PbMut may be lower than that of PbMut-P91. The difference in variability and fitness of each library may have led to distinct sets of mutants being isolated. It is reported that parasite clones possessing mutations in *P. falciparum acetyl-CoA transporter* (*pfact*) and *UDP-galactose transporter* (*pfugt*) gene exhibit resistance against the antimalarial imidazolopiperazines KAF156 and GNF179. The mutants were only isolated through cloning immediately after drug exposure not by conventional long-term drug pressure, to avoid being outcompeted by fitter mutants (30), thus highlighting the importance of mutant fitness in screening outcomes. Therefore, the identification of disparate sets of SNPs in our two screening studies may be attributed to the fitness and/or potential variability of the mutants present in each library.

Since our aim was to isolate PPQ-resistant parasites by applying PPQ pressure, we focused on the clones from PbMut-P6, the parasite population obtained by the final screening, as these were expected to exhibit the strongest resistance phenotypes. As a result, we isolated clones with reduced PPQ susceptibility and identified five SNPs that were conserved among these clones. Out of the five identified SNPs, three were detected in *zinc carboxy peptidase*, s*porozoite invasion-associated protein 1 (SIAP-1)*, and *NAD(P) transhydrogenase*, which are exclusively expressed within mosquito stages according to the transcriptome data in PlasmoDB (https://plasmodb.org/plasmo/app). Previous studies have shown that disruption of *SIAP-1* and *NAD(P) transhydrogenase* results in defects in the sporozoite stages (24, 25), suggesting that their biological roles are during transmission through the mosquito. It is therefore plausible that these three mutations are not associated with PPQ resistance within asexual stages. The remaining SNPs were found in a *conserved Plasmodium protein* and *chloroquine resistance transporter,* which are constitutively expressed throughout the parasite life cycle according to the transcriptome data in PlasmoDB. This suggests that the mutations in these genes may be associated with PPQ resistance. Further investigations are required to address the association between these mutations and PPQ susceptibility.

We chose to investigate PbCRT Y119* because the association of PbCRT N331I to reduced PPQ susceptibility was demonstrated in our previous study (23). The anticipated structure of PbCRT suggests that the stop codon in PbCRT Y119* corresponds to the cystolic face of the WT PbCRT protein immediately following the second TM domain. Such a drastic truncation of PbCRT would likely render it non-functional—contrary to the supposition that PbCRT is indispensable for parasite survival, based on two unsuccessful attempts to disrupt it using conventional gene replacement strategies (27, 28). Our attempts using the same strategy were similarly unsuccessful. Therefore, to further assess its essentiality, in the present study, we utilized genome editing methodology and successfully deleted the entire *PbCRT* gene. The previous attempts to replace *PbCRT* with a larger gene fragment such as a selectable marker may have interfered with the proper expression of neighboring genes, potentially leading to lethality in the recombinant parasites. Alternatively, *PbCRT*(−) parasites produced by the replacement strategy may incur a lower fitness and are outcompeted by PbWT parasites.

Given the non-essentiality of *PbCRT*, we attempted to insert a stop codon at the corresponding position in *P. falciparum (PfCRT* R122*) by genome editing. Five attempts were unsuccessful, suggesting that *PfCRT* is indeed essential. This is supported by the fact that a broad study employing the random insertion of piggyBac transposons in *P. falciparum* indicated that *PfCRT* appeared to be intractable to manipulation (31). In addition, nonsense mutations within *PfCRT* have not been detected in the 3,488 *P. falciparum* genomes that are now available from 23 countries (32). One report described two clinical parasite isolates from Sudan that express a premature splice variant of *PfCRT* mRNA; however, the impact of such a shorter *PfCRT* mRNA on the parasite survival seems to be minimal because mature *PfCRT* mRNAs were also transcribed in these parasites (33). Overall, there is no evidence supporting the dispensability of *PfCRT*; thus, to date, the non-essential feature of CRT is only observed in *P. berghei*.

In *P. falciparum*, it has been reported that *PfCRT* knock-down parasites accumulate peptides that are 2 to 8 amino acids long in the DV (29). Similarly, it has been reported that PfCRT transports peptides that are 4 to 11 amino acids long in the *Xenopus* oocyte expression system (34). Collectively, these findings suggest that oligopeptides produced by hemoglobin degradation in the DV are exported by PfCRT to the cytosol, where they are further digested and used for protein synthesis. Additionally, the *PfCRT* knock-down parasites showed a more severe defect in the blood stages and a swollen DV, suggesting that the export of oligopeptides by PfCRT may contribute to maintaining the colloid osmotic balance in the DV (29). In the present study, we do not have data that suggest the natural function of PbCRT, although we observed comparable morphological abnormalities and fitness costs on *PbCRT*(−) parasites, suggesting similar roles of PbCRT and PfCRT. We conducted a phenotypic analysis of *PbCRT*(−) parasites during the mosquito stages. The number of oocysts in the midgut of mosquitoes infected with *PbCRT*(−) parasites was significantly lower than that of PbWT parasites despite equal numbers of gametocytes from *PbCRT*(−) and PbWT being fed to mosquitoes. This finding strongly indicates that PbCRT plays a role in oocyst formation that is independent of hemozoin production. Further investigations revealed that the number of sporozoites in the salivary glands and the transmission efficiency of *PbCRT*(−) parasites were both reduced compared to PbWT. This reduction could be attributed to the lower number of oocysts, subsequently leading to fewer sporozoites in the salivary glands and, consequently, fewer sporozoites being injected into the host during transmission. Alternatively, the absence of PbCRT may impose fitness costs specifically on the transmission stages. It is important to note that the assessment of fitness cost on transmission efficiency via natural feeding has certain limitations. Nonetheless, our comprehensive phenotypic study across the parasite's life cycle clearly demonstrates that, while PbCRT is not essential for parasite survival, its absence imposes a significant fitness cost, particularly affecting blood stages and oocyst formation. These findings imply that other molecular components may partially compensate for the loss of PbCRT in *PbCRT*(−) parasites, allowing them to maintain viability.

It is known that *P. falciparum* acquires drug resistance through non-synonymous mutations in certain genes, such as *PfCRT*, *DHFR-ts*, and *DHPS*, as well as an increase in copy number of *PfMDR1* and *plasmepsin II* and *III*, resulting in an alteration or increase in the activity of each gene product (35). This mode of drug resistance acquisition is termed a gain of function. Alternatively, parasite drug resistance acquisition can occur through a decline in the function of gene products. For example, a mutation in the *kelch13* gene confers artemisinin resistance by reducing hemoglobin uptake activity (36, 37). The aforementioned mutations occur in genes essential for *P. falciparum* survival whereas, in contrast, we show that *PbCRT* is a non-essential gene and that *PbCRT*(−) represents a complete loss of function. Such examples have been only reported for mutations in *Pfact* and its *P. berghei* orthologous gene, *Pbact*, through screening with imidazolopiperazine. Mutants exhibiting resistance against this drug harbor multiple stop codons in these genes (30), suggesting that the gene products may lose function. It is conceivable that the parasite may have acquired a drug-resistant phenotype by deleting the relevant drug resistance molecule such as PbCRT at the expense of fitness, while some molecules may partially compensate for PbCRT function and confers drug resistance phenotype to the parasite. Further study is required to address the molecular mechanisms by which the functional disruption of these non-essential gene products contributes to drug resistance.

In this study, we found that *PbCRT* is a non-essential gene, which contrasts with the essential nature of *PfCRT* in *P. falciparum.* Further characterization of the *PbCRT*(−) parasite confirms that the absence of *PbCRT* leads to reduced susceptibility to PPQ. Currently, most studies on drug resistance mechanisms have focused on non-synonymous mutation and copy number elevation in essential genes. In this context, further investigation into the association between nonsense mutations in non-essential genes, such as *PbCRT* and reduced PPQ susceptibility, could reveal novel drug resistance mechanisms. This could also lead to the identification of new drug targets that act through mechanisms distinct from those conferred by mutations in essential genes.

## MATERIALS AND METHODS

### Animals and parasites

Female mice (ddY and Balb/c) aged 5–6 weeks (Sankyo Labo Service Corp., Japan) were used in this study. *Anopheles stephensi* mosquitoes were reared as previously described (38, 39). *Plasmodium berghei* ANKA (clone 2.34) was used to generate transgenic parasites.

A marker-free mutator *P. berghei* parasite line utilizing CRISPR/Cas9 was generated; specifically, a donor DNA possessing two amino acid mutations (D311A and E313A) critical for the proofreading activity of DNA polymerase δ and corresponding sgRNA were inserted into the pBC-1 plasmid (39). The subsequent parasite transfection and cloning were conducted as previously described (40). This transgenic parasite is referred to as PbMut.

The *P. falciparum* 3D7 strain was a kind gift from the National Institute of Allergy and Infectious Diseases and was cultured as previously described (41) with type-O human erythrocytes at a hematocrit of 2% in RPMI 1640 media (Thermo Fisher Scientific, Cat. No. 23400–021, USA) and supplemented with 5% type-AB human serum, 0.25% Albumax II (Thermo Fisher Scientific, Cat. No. 11021029, USA), 200 mM hypoxanthine (Sigma-Aldrich, Cat. No. H9377, USA), 2% (wt/vol) sodium bicarbonate (Sigma-Aldrich, Cat. No. S5761, USA), and 10 mg/mL gentamicin (Thermo Fisher Scientific, Cat. No. 15750060, USA). The parasite was cultured under a gas mixture of 5% CO_2_, 5% O_2_, and 90% N_2_.

### Isolation of piperaquine (PPQ)-resistant parasites from the PbMut line

**Fig 6:**
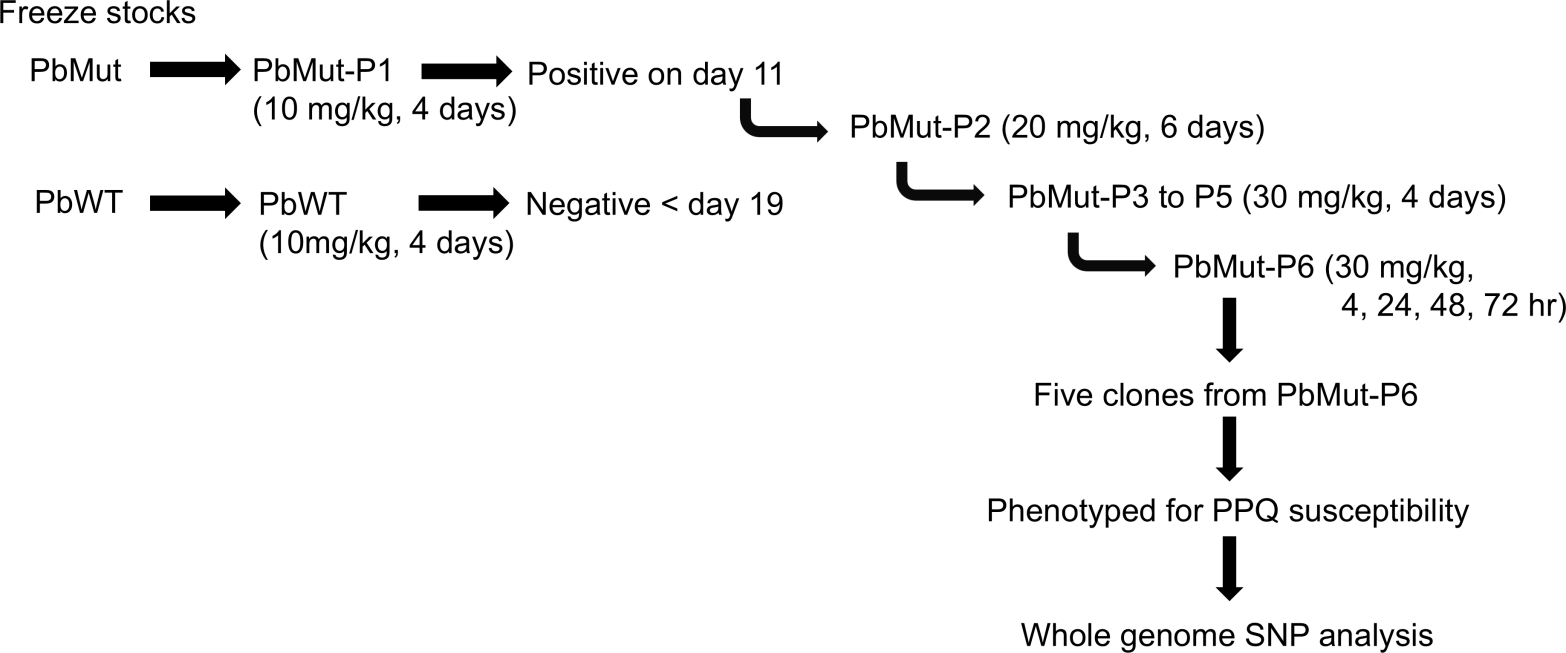
Flow chart of six cycles of screening for PPQ-resistant parasites. Frozen stocks of PbMut and PbWT were inoculated into mice (PbMut-P1 and PbWT, respectively) and subjected to 4 days of PPQ treatment (10 mg/kg). Parasite patency was confirmed in PbMut-P1-infected mice on day 11 but was not observed in PbWT until day 19. The infected blood of PbMut-P1 (1 × 10^5^) was serially passaged through PbMut-P2 to -P6 under PPQ pressure. Five clones isolated from PbMut-P6 were characterized for phenotype and subjected to SNP analysis.

The procedure for the screening for drug-resistant parasites using the PbMut parasite line is presented in Fig. 6. Briefly, frozen stocks of infected red blood cells (iRBC) from PbMut or wild type (PbWT) were inoculated into mice designated as PbMut-P1 (passage 1) and PbWT, respectively. On day 3 after parasite inoculation, PPQ tetraphosphate tetrahydrate (Sigma-Aldrich, Cat. No. C7874, USA) was administered intraperitoneally daily for 4 days, and the parasitemias were monitored until days 12 and 19 for PbMut-P1 and PbWT, respectively. When the parasitemia reached 1%, iRBC (1 × 10^5^) of PbMut-P1 were injected into a new mouse (PbMut-P2), followed by repetition of the PPQ administration and serial passages to new mice. At the last round of drug pressure, the mice infected with PbMut-P5 and PbWT were denoted as PbMut-P6 (passage 6) and PbWT, respectively, and were treated with PPQ at 4, 24, 48, 72, and 96 h post-infection. For PPQ doses, 10, 20, and 30 mg/kg were used for PbMut-P1, PbMut-P2, and PbMut-P3 to -P6, respectively. For the parasite cloning, iRBC were taken from PbMut-P6 on day 3 and were used for limiting dilution cloning to isolate five clones, named PbMut-P6-C1 to -C5. To evaluate the PPQ susceptibility, iRBC (1 × 10^5^) from the five isolated clones, PbMut-P6, and two clones of PbWT were each injected intravenously into mice and then subjected to PPQ treatment (30 mg/kg) at 4, 24, 48, and 72 h post-infection in a 4-day suppressive test (42). Parasitemias were monitored until day 8 and were calculated by counting over 2,000 RBC on Giemsa-stained slides under a light microscope (magnification, ×1,000). If no iRBC were detected, an additional 3,000 RBC were checked. The susceptibility to PPQ of the parental PbMut-P6 strain and its clones was evaluated following parasite recovery after over 3 months of storage at −80°C (Table S3).

### Whole-genome sequencing and single nucleotide polymorphism (SNP) analysis

Genomic DNA was extracted from the leukocyte-removed blood of mice infected with PbMut-P6 clones and sequenced using a DNBSEQ-G400 system (MGI Tech Co. Ltd., China). CLC Genomics Workbench ver. 11 (QIAGEN, Germany) was used to perform whole genome SNP analysis, with default parameters for mapping reads to the reference genome of *P. berghei* ANKA (PlasmoDB-60). Multigene family genes were excluded from the SNP analysis to prevent read mismappings. SNPs were extracted using Basic Variant Detection in CLC-GW, following a threshold of over 10 reads and 80% frequency. We used SNPs from the PbMut-P91 clone, which had been serially passed through mice until P91 under drug-free conditions, as a control (23). PbMut-P91 is expected to contain many mutations unrelated to PPQ resistance and, therefore, can serve as a stringent filter to exclude SNPs not associated with PPQ resistance from the individual clone's SNPs. Based on this assumption, the SNPs of the PbMut-P91 control were excluded from the consideration of SNPs within the PbMut-P6 clones. The remaining non-synonymous and nonsense SNPs that were conserved in all clones were extracted as candidates for PPQ-resistant associated SNPs.

### Generation of *PbCRT*(−) and *PfCRT* R122* parasites

For the generation of *PbCRT* gene knockout [*PbCRT*(−)] parasites, two DNA fragments corresponding to the 5′- and 3′-untranslated regions of the *PbCRT* gene were separately amplified using the specific primer pairs 5′UTR-F/5′UTR-R and 3′UTR-F/3′UTR-R, respectively. The two PCR products were joined using overlap PCR and primers 5′UTR-F/3′UTR-R. The resulting 1.6-kb PCR product was ligated into the HindIII/AflII sites of pBC-1 using an In-Fusion HD Cloning kit (TAKARA BIO Inc., Japan). A guide RNA (gRNA) was generated by annealing gRNA-F1 and -R1, which covers −15 to +5 of the *PbCRT* gene, followed by ligation into the BsmBI site of pBC-1. The final plasmid, pPbCRT-KO, was then electroporated into wild-type *P. berghei*, followed by parasite cloning as previously described (40). Genomic DNA was extracted from a *PbCRT*(−) parasite clone and subjected to diagnostic PCR using the primer pair P1-F/P1-R. The complete deletion of the *PbCRT* gene in the *PbCRT*(−) parasite clone was confirmed by Sanger sequencing of the PCR product and also by whole-genome sequencing of a *PbCRT*(−) parasite clone. The reads that mapped to the region surrounding the *PbCRT* gene were generated and visualized using the Integrative Genomic Viewer (IGV) developed by the Broad Institute.

Transgenic *P. falciparum* parasites possessing a stop codon at amino acid 122 in PfCRT (PfCRT R122*) were generated as previously described (41). In brief, a linear donor DNA (*PfCRT* R122*) was generated by PCR to create two products using primers PfCRT5′UTR-F/-R and PfCRT3′UTR-F/-R, which were then used as a template to overlap by PCR using primers PfCRT5′UTR-F/PfCRT3′UTR-R. Two 20-bp sgRNA target sequences were designed using the CHOPCHOP program (https://chopchop.cbu.uib.no/). The plasmid coding Cas9 and sgRNA1 and the linear donor DNA were mixed and were used to transfect *P. falciparum* schizonts using a four-dimensional nucleofector (Lonza, Swiss). One day after the transfection, 25 ng/mL pyrimethamine (MP Biomedical, Cat. No. 194180, USA) pressure was applied to the parasites for 10 days, and the parasite culture was continued another week without the drug. Genomic DNA was then extracted from the parasite population, and the targeting region was PCR-amplified and subjected to direct Sanger sequencing. The primers used in this study are listed in Table S4.

### Evaluation of parasite fitness in mice and mosquitoes and transmission efficiency

To compare the growth rates of *PbCRT*(−) and PbWT in mice, iRBC (1 × 10^5^) of each parasite line were intravenously injected into respective groups of three mice each, and the parasitemias were monitored up to day 7. *PbCRT*(−) parasite fitness in the mosquito and subsequent transmission to mice were evaluated as previously described (38). In brief, mice harboring similar *PbCRT*(−) or PbWT gametocytaemias were used to feed 4- to 7-day-old female mosquitoes. Fully engorged mosquitoes were collected, and the oocyst prevalence, the number of oocysts per midgut, and sporozoites per salivary gland were quantified on days 10 and 18 post-blood feeding, respectively. Mosquitoes carrying *PbCRT*(−) and PbWT sporozoite parasites were then fed on BALB/c mice on day 20 post-blood feeding. Parasite transmission from mosquitoes to mice and the prepatent period were examined by checking blood smears for two weeks after feeding.

### Association of *PbCRT* deletion and PPQ susceptibility

For the comparisons of PPQ efficacies, iRBC (1 × 10^5^) from *PbCRT*(−), PbMut-P6-C1, and PbWT parasite lines were intravenously inoculated to respective groups of three mice each, which were then treated with PPQ (30 mg/kg) at 4, 24, 48, and 72 h post-infection, and the parasitemias were monitored until day 7.

### Software and statistical analysis

The prediction of transmembrane domains in PbCRT was performed using DeepTMHMM (https://dtu.biolib.com/DeepTMHMM). Statistical analysis was performed using GraphPad Prism 10. Comparisons of the parasitemias between two independent groups were made by the Student's *t*-test. The parasitemias were expressed as means and standard deviations. The numbers of midgut oocysts and salivary gland sporozoites were analyzed by the Mann-Whitney *U* test. The prepatent periods of mice challenged by PbCRT(−) and PbWT were analyzed by Welch's test, and *P* < 0.05 was considered statistically significant.

## Data Availability

The *P. berghei* genome sequences of PbMut-P6 clones have been deposited in DDBJ with the accession number DRA015256.

## SUPPLEMENTAL MATERIAL

Supplemental tables aac.01589-24-s0001.xlsx Tables S1 to S4.

ASM does not own the copyrights to Supplemental Material that may be linked to, or accessed through, an article. The authors have granted ASM a non-exclusive, world-wide license to publish the Supplemental Material files. Please contact the corresponding author directly for reuse.
